# Study on the needs of the older adults with dementia in care institutions based on a hierarchical interaction model: a case study of an institution in Hangzhou, China

**DOI:** 10.3389/fpubh.2025.1633806

**Published:** 2025-09-16

**Authors:** Hong Sun, Rui Chai, Song Huang, Wen Xue, Zhenhua Wang, Xinnan Yu, Zijun Zhu

**Affiliations:** ^1^College of Urban Construction, Zhejiang Shuren University, Hangzhou, China; ^2^College of Architecture and Art, Hebei University of Architecture, Zhangjiakou, China; ^3^Chinese Academy of Sciences Institute of Tibetan Plateau Research, Beijing, China

**Keywords:** dementia care, needs assessment, Maslow’s hierarchy of needs, person-centered care, cultural context difference, intervention strategies

## Abstract

Dementia care is a significant public health challenge, particularly in institutional settings where standardized management often conflicts with individual needs. This research investigates the multifaceted needs of older adults with dementia in a Chinese care institution and the extent to which these needs are met. By constructing a stratified interactive framework for need analysis and employing methods such as field observations, in-depth interviews, and surveys, this study comprehensively assessed the satisfaction levels of residents’ multidimensional needs. The findings indicate that while the physiological and safety needs of the residents were largely met, a significant deficit existed in their needs for love and belonging. Furthermore, the Eastern cultural context, with its emphasis on family and collective identity, also influenced the prioritization of needs among this population. Consequently, this study concludes that there is an urgent need to strengthen family interaction and personalized care and proposes a series of targeted interventions, offering theoretical and practical insights for the advancement of person-centered and precise institutional dementia care.

## 1 Introduction

The accelerating process of global aging and the sustained increase in dementia prevalence (1) have positioned dementia care as a significant public health challenge in the 21st century (2). According to the World Health Organization, as of 2023, the global number of individuals with dementia has surpassed 55 million, with China accounting for more than a quarter of this population. Furthermore, this number is projected to double every 20 years (3). In China, although specialized dementia care institutions—characterized by certified qualifications and dedicated care units—have become a mainstream choice due to their standardized services (4), the conflict between standardized management and individual needs is becoming increasingly prominent (5): A safety-focused collective care model frequently limits residents’ autonomy (6), while delays in the identification of personal needs aggravate agitated behaviors (e.g., wandering, resisting care) (7).

Existing theoretical frameworks predominantly emphasize static need stratification (8), which limits their capacity to comprehensively explain the complex behavioral mechanisms of individuals with dementia (9). For instance, although Maslow’s Hierarchy of Needs offers a classical basis for need classification, it struggles to account for the coexistence of multi-level needs caused by neurodegenerative disorders (10), as well as the non-linear patterns of need expression (11). Historically, researchers have explored the application of Maslow’s Hierarchy of Needs in dementia care to construct more comprehensive need assessment models. Notably, Jongsma et al. (12) introduced the Hierarchy Model of Needs in Dementia, emphasizing the relationship between unmet needs and health-related quality of life.

Although the Need-Driven Dementia-Compromised Behavior (NDB) model has elucidated behavioral compensation mechanisms via the “background-proximal” dual-factor framework (13), it insufficiently integrated cultural and socio-psychosocial factors (14). Concurrently, Kitwood’s “Person-Centered Care Theory” emphasizes maintaining the patient’s personality through identity activation and the maintenance of social connections (15), but the practical application of its intervention strategies lacks the support of a dynamic need model. Recent evidence has shown that in an Eastern cultural context, dementia patients tend to seek group belonging rather than individual expression to meet their needs (16), which indicates the necessity for culturally adapted interventions. However, relevant empirical research remains limited.

This study integrates Maslow’s Hierarchy of Needs, the NDB model, and Kitwood’s Person-Centered Care Theory to establish a “stratified-Interactive Needs Analysis Framework” (Table 1). This framework employs Maslow’s hierarchy as the classification basis to identify the non-linear and coexisting nature of dementia patients’ multi-dimensional needs. The NDB model is utilized to examine the dynamic mechanisms through which needs are transformed into specific behaviors (e.g., wandering, resistance to care) driven by background and proximal factors. Additionally, Kitwood’s theory is incorporated as an intervention modulation variable, employing strategies such as identity awakening and social reinforcement to break the “need suppression → behavioral compensation” cycle (17), thereby forming a “classification-interpretation-intervention” closed-loop system. In the synergy of the three, the NDB model endows Maslow’s static hierarchy with dynamic interpretive capacity, elucidating “why needs are transformed into behaviors,” while Kitwood’s theory offers practical transformation tools (such as vocational reenactment activities), guiding “how to address unmet needs,” and seeking possible paths to optimize the care model through multi-dimensional intervention (18). This framework provides both theoretical rationale and practical guidance for building a person-centered care system that better aligns with the needs and cultural characteristics of the older adults with dementia (19). achieving a cohesive integration of theory and practice.

**Table 1:** Stratified-interactive needs analysis framework.

Theory	Maslow’s hierarchy of needs	NDB model dimensions	Kitwood intervention strategy
Function	Basis for need classification	Mechanism of behavior transformation	Variable for intervention adjustment
Interaction	Provides need identification criteria for the NDB model and indicates the target hierarchy for Kitwood’s intervention strategy.	Transforms Maslow’s needs into observable behaviors, revealing compensation pathways for suppressed needs; provides mechanistic explanations for Kitwood interventions.	Compensates for the NDB model’s neglect of cultural and social psychology by addressing gaps in Maslow’s needs through practical intervention strategies.

## 2 Method

### 2.1 Research subjects

The field investigaion was conducted at a professional dementia care facility located in Hangzhou, Zhejiang Province, China. The designated care zone comprises 8 floors, with each functioning as an autonomous care unit, accommodating a total of 108 older adults with varying degrees of cognitive impairment. This study began on January 10, 2025 and ended on January 25, 2025. This study was approved and funded by four related funds, and has been approved by the College of Urban Construction, Zhejiang Shuren University, with documented permission from the research subjects’ institutions.

This study began on January 10, 2025, and ended on January 25, 2025. To ensure the smooth conduct of the study and to build trust with the older adults with dementia, three researchers initiated preliminary engagement by conducting on-site visits twice a week, beginning December 13, 2024, prior to the team’s formal entry into the institution on January 10, 2025. This preliminary phase allowed the researchers to establish a friendly rapport with the residents. Furthermore, throughout the formal research period (January 10–25), our data collection efforts, including observations and interviews, were conducted with the necessary support and assistance of the institution’s professional care staff and managers. This collaborative approach not only ensured the ethical and safe conduct of the study but also greatly facilitated effective communication with the research participants.

All research participants possessed the capacity to provide informed consent and signed the corresponding consent forms; the older adults lacking this capacity were excluded from the study. Throughout the research process, including interview and questionnaire sessions, professional care staff were present to provide assistance and oversight. If a participant exhibited significant fluctuations in their psychological or physiological state, rendering them unable to continue, the session was promptly paused and was only resumed once their condition had stabilized. The measures were implemented to ensure that all participants maintained their physical and psychological well-being and retained full capacity for informed consent throughout the study.

Major observations and interviews were conducted with the older adults with dementia and supplementary interviews were conducted with caregivers and institutional managers. The inclusion criteria for the interviewed dementia patients are as follows:

1. Aged 60 years or older (in accordance with the World Health Organization’s definition of older persons);
2. Having resided in the institution for six months or more;
3. Scoring 1 to 2.5 on the Cognitive Dementia Rating Scale (CDR) (mild to moderately severe dementia);
4. Having a certain language expression ability and being able to complete at least a small part of communication;
5. Retaining basic non-verbal communication abilities (such as gestures and facial expressions);
6. Exhibiting observable agitated behaviors (e.g., resistance to care, wandering);
7. Voluntarily participating in this study.

Exclusion criteria (for dementia patients):

1. Voluntary withdrawal from the study at any point during observations or interviews.
2. Inability to maintain a reasonably stable physiological and psychological state.
3. Inability to maintain the capacity for informed consent throughout the research process.

A total of 30 eligible the older adults were ultimately included (Table 2). Among them, the proportion of those with mild to moderate dementia was 93% (CDR = 1–2), and the proportion of those with moderately severe dementia was 7% (CDR = 2.5), consistenting with the research objective.

**Table 2:** Basic information of observed and interviewed dementia patients.

Variable	Categories	Frequency/No.	Percentage (%)
Gender	Male	16	53
	Female	14	47
Age	≤ 60	1	3
	61–70	5	17
	71–80	9	30
	81–90	12	40
	≥ 91	3	10
Dementia	Moderate to severe dementia	2	7
	Moderate dementia	9	30
	Mild to moderate dementia	10	33
	Mild dementia	9	30

Inclusion criteria for the caregivers are as follows:

1. Having been employed at the institution for six months or more;
2. Currently engaged in diagnostic, caregiving, or management tasks associated with dementia patients;
3. Capable of conducting effective communication;
4. Voluntarily participating in the study and obtaining approval from the institution.

Exclusion criterion: Withdrawal from the- study during the observation or interview period. Ultimately, 20 professional caregivers were included (Table 3).

**Table 3:** Demographic characteristics of interviewed caregivers.

Variable	Categories	Frequency/No.	Percentage
Gender	Male	9	45
	Female	11	55
Age	26–30	3	15
	41–50	8	40
	51–60	9	45
Professional background	Professional caregiver	2	10
	General caregiver	15	75
	Healthcare-related worker	1	5
	Other	2	10

Work background classification is based on whether caregivers possess relevant professional certifications.

### 2.2 Data collection protocol

After obtaining the informed consent from the relevant personnel and the research subjects, the researcher entered the target institution as a “campus volunteer” to conduct the fieldwork. Data were collected through triangulation method (20): semi-participatory observation (8 h daily) in combination with semi-structured interviews was implemented for the older adults with dementia, and structured interviews with participant observation were adopted for caregivers.

The study employs an exploratory sequential mixed-methods design (21), and systematically carried out in three phases:

Phase 1 - Observational Study: Researchers maintained a rigorous position of an observer as an outsider (22), and established research trust through a gradual contact strategy to foster research trust. Behavioral patterns of dementia patients during daily activities were meticulously documented to complete baseline data collection.

Phase 2 - Contextual Evaluation: Contextual interviews and standardized scale measurements were implemented with two components:

(1) Semi-structured interviews were conducted with the older adults with dementia. Standardized need satisfaction scales covering five dimensions such as physical, safety, and belonging were collected in different life situations (5-level Likert scale), and around the “behavior - need” correlation, special or meaningful phenomena observed were further inquired (such as “Why are you always walking in the corridor?”), The semi-structured interview protocol is presented in Table 4.

**Table 4:** Semi-structured interview questionnaire for the older adults with dementia.

No.	Question
1	Gender / Age
2	Previous Living and Care Arrangement: (e.g., Did your children participate in your care? Did you live alone?)
3	Transition and Adaptation: What has it been like for you to live here after leaving your home?
4	Perception of Care: In your opinion, what are the main differences between the care you receive here and the care you received at home?
5	Daily Comfort: How do you feel about the convenience and comfort of your daily meals, activities, and rest periods?
6	Unmet Physical Needs: Have there been times when your physical needs were not met in a timely manner (e.g., feeling thirsty, too hot or cold, needing to use the restroom)?
7	Environmental Safety: Do you feel safe in this environment? Have you ever worried about getting injured or lost?
8	Social Connection: How would you describe your relationships with others here, including other residents and the caregivers? Do you feel a sense of closeness?
9	Family Connection: How often do your family members or relatives visit you? Do you miss them?
10	Social Engagement: Do you take the initiative to talk to others or participate in group activities? Are there any activities that you particularly enjoy?
11	Respect and Dignity: Do you feel that the caregivers respect your personal habits and preferences (e.g., using respectful titles like ‘Teacher’ or ‘Comrade,’ protecting your privacy)?
12	Unfulfilled Aspirations: Is there anything you would particularly like to do but are currently unable to? What are the reasons for this?
13	Activity Engagement: Does the staff or the institution arrange activities or tasks that you personally enjoy? Are you willing to participate in them?
14	Negative Events: Can you recall any specific incident that made you feel particularly unhappy or wronged?
15	Emotional Expression: When you feel angry or unhappy, how do you typically express your emotions?

(2) Semi-structured interviews with caregivers were also performed to investigate caregiving challenges. Transcribed audio data were subsequently encoded using Nvivo12 software.

Phase 3 - Theoretical saturation examinations were conducted on the data from the initial interviews and observations, alongside supplementary data acquisition, to ensure the comprehensive development of core themes and categories (23). After initially coding 28 valid interview transcripts, the research team assessed the main categories for saturation based on the dual criteria of ‘no new concepts’ and ‘no new properties’. Theoretical saturation was deemed to have been reached when no new concepts emerged from subsequent data, and the characteristics, scope, and relationships of each core category had been sufficiently captured.

The fieldwork schedule was subordinate to the daily routines of the older adults with dementia and the working hours of caregivers. Data collection methods included experiential field notes, audio recordings, photographs, structured questionnaires, etc. The fieldwork strategies, questionnaires, and interview protocols were dynamically adapted according to daily data feedback.

To safeguard the privacy of the older adults with dementia and adhere to research ethics standards (in accordance with Article 73 of China’s Personal Information Protection Law and the World Health Organization’s ethical principles for health research), all collected data was subjected to a triple anonymization process: voice distortion (eliminating identifying characteristics by shifting the voiceprint’s fundamental frequency), facial blurring (obscuring biological identifiers with mosaics), and de-identification of personal information (removing real names, addresses, and institutional codes, and replacing them with research identifiers such as “5F1”).

## 3 Results

### 3.1 Quantitative analysis

The quantitative data obtained from field surveys were systematically inputted into SPSS software. Based on the five dimensions of Maslow’s Hierarchy of Needs Theory, a quantitative analysis of the degree of need satisfaction among the older adults with dementia was conducted. In combination with interviews and field observations, the satisfaction of actual needs in institutional care was specifically explored. The data were evaluated using a 5-point Likert scale (1 = strongly disagree, 5 = strongly agree), and the structural validity of each dimension was confirmed through principal component analysis. The results of the reliability and validity assessments demonstrated that the Cronbach’s *α* coefficients of each dimension ranged from 0.71 to 0.83, while the composite reliability (CR) values were all above 0.7, all conforming to the Fornell-Larcker criterion (24).

To enable a nuanced interpretation of the data in our quantitative analysis, we established three core evaluation metrics for each question: ‘Agreement,’ ‘Mode,’ and ‘Mean Score.’ The Mean Score reflects the overall trend of need satisfaction; the Mode reveals the most frequent respondent attitude, mitigating the effect of outliers on the mean; and ‘Agreement’—defined as the percentage of respondents selecting 4 (‘Agree’) or 5 (‘Strongly Agree’) on the 5-point Likert scale—directly measures the size of the group holding a positive attitude of satisfaction. By integrating these three metrics, we can more precisely identify ‘implicit need gaps’: situations where a state of being ‘not opposed, but not truly satisfied’ is prevalent.

#### 3.1.1 Physiological needs

With the dementia progresses, the basic physiological functions of the older adults gradually deteriorate, such as weakened activity capabilities in aspects like eating, excretion, and dressing and undressing. At this time, timely recognition and assistance from caregivers are particularly critical (25). The physiological needs scale comprises 14 measurement items. Analyzed by SPSS, the standardized reliability coefficient is 0.725, and the validity coefficient is 0.743, all conforming to the Fornell-Larcker standard. Relevant data are detailed in Table 5.

**Table 5:** Quantitative evaluation of physiological needs fulfillment.

No.	Question	Agreement	Mode	Mean score
1	The institution’s schedule is well-suited to your physical condition.	20	3	2.57
2	The institution provides a quiet and comfortable sleeping environment.	27	3	3.21
3	The meals provided are nutritionally balanced and easy to digest.	63	4	3.77
4	The institution reminds you to eat on time.	73	5	4.07
5	The variety of food options is sufficient and can be adjusted according to your preferences.	60	5	3.77
6	The assistive eating tools provided are suitable for your use.	23	3	2.43
7	Caregivers assist you with eating when necessary.	63	5	3.9
8	You are able to drink enough water every day.	67	5	4
9	Caregivers take care of your skin health and prevent pressure ulcers.	83	4	4.17
10	Caregivers provide timely assistance when you need to change clothes or get dressed.	63	5	3.77
11	Caregivers help you with bathing and other personal hygiene needs.	73	4	4.03
12	Caregivers monitor your physical health and provide necessary support (e.g., oral and nasal care, vision support).	50	3	3.63
13	The institution provides appropriate assistive walking devices for mobility support.	15	3	2.6
14	The institution adjusts indoor temperature and humidity to enhance comfort.	24	3	3.23

‘Agreement’ refers to the percentage of respondents who rated an item with a score higher than 3 (i.e., a score of 4 or 5) on the 5-point Likert scale.

The overall data show that “basic support is more than enough, but personalized comfort is not enough.” Higher scores on basic items such as question 3 (73% agreement; mean score of 4.07) and question 11 (73% agreement; mean score of 4.03) indicate that standardized physiological support is provided. Integrating questionnaire data and interview insights, a comprehensive analysis was performed on items with a low degree of recognition (<50%) and low average scores (<3):

1. Daily Routine: Due to the large number of the older adults within the institution and the complexity of management, it is challenging to develop individualized schedules for each elder. Nevertheless, a unified daily routine is beneficial for facilitating social interaction and collective activities.
2. Resting Environment: Although the overall approval rate for this item is 27%, it reveals considerable internal variations. The majority of the older adults consider the night-time resting environment to be relatively satisfactory, while they are significantly disturbed by noise during the midday nap.
3. Walking Assistance: Older adults residents mainly depend on family-provided devices, as the institution lacks personalized mobility assistive devices (e.g., non-slip handles, height-adjustable devices), thereby constraining their independent mobility.
4. Temperature and humidity environment: Although central air conditioning is available, the building’s extensive layout and the seasonal variability in gabled areas, coupled with the absence of dedicated humidity control facilities, impose challenges on effective temperature and humidity regulation.
5. Diet: While most the older adults receive comprehensive feeding support, the absence of dementia-specific dining utensils and furniture, along with a shortage of caregivers, results in delayed responses to nuanced care needs.

#### 3.1.2 Security requirements

Despite residing in a professional dementia care institution, primarily due to cognitive decline, memory deficits, and emotional instability, the older adults continue to face physiological risks, including accidental injuries and challenges related to medication management (25). Simultaneously, they require a stable environment and consistent psychological support to mitigate internal feelings of insecurity and fear (26). The Safety Needs Scale consists of 10 measurement items, with items 1–4 representing the psychological safety dimension and items 5–10 reflecting the physiological safety dimension. The SPSS analysis results demonstrate that the standardized reliability coefficient of this scale is 0.832 and the validity coefficient is 0.726, indicating satisfactory reliability and validity. The detailed data are presented in Table 6.

**Table 6:** Quantitative evaluation of safety needs fulfillment.

No.	Question	Agreement	Mode	Mean score
1	The presence of other residents’ family members or volunteers makes you feel uneasy.	7	1	2.1
2	A regular schedule and structured activities provide you with a sense of stability.	57	3	3.73
3	The institution regularly assesses your psychological state and provides appropriate support.	24	3	2.43
4	You do not feel anxious about the decline in your physical functions.	32	3	2.87
5	The institution’s fall prevention and safe mobility measures make you feel secure.	50	4	3.57
6	Regular health check-ups and monitoring give you and your family peace of mind.	50	3	3.63
7	Caregivers respond promptly to any sudden health issues.	70	4	3.83
8	The institution’s measures to prevent wandering (e.g., gated entrances) ensure your safety.	54	4	3.73
9	The precise medication management by caregivers makes you feel reassured.	73	5	3.97
10	The institution implements strict infection prevention measures to ensure your health and safety.	73	5	4.13

‘Agreement’ refers to the percentage of respondents who rated an item with a score higher than 3 (i.e., a score of 4 or 5) on the 5-point Likert scale.

The data from the table vividly illustrates an imbalance: physiological safety needs are met, whereas psychological safety needs are deficient. The scores for physiological safety items (Questions 5–10), including agreement rates and mean scores, are relatively high, indicating that the institution effectively ensures the residents’ physical safety through rigorous management protocols. In contrast, the deficiency in the psychological safety dimension is markedly more pronounced. Specifically, the low scores for Question 3 (Agreement: 24%; Mean Score: 2.43) directly point to the institution’s lack of a comprehensive psychological assessment and comfort system. Consequently, the negative emotions arising from factors such as aging and illness (27) are not effectively addressed or managed.

#### 3.1.3 Belongingness and love needs

Following the admission to care facilities, older adults with dementia frequently experience emotional deprivation stemming from prolonged separation from their families, and they have a pronounced need for companionship and emotional support (28). In their daily lives, they mainly depend on interactions with other residents and caregivers to establish a sense of belonging (29). Furthermore, institutions often organize communication sessions, festive celebrations, and similar activities to provide residents with direct and accessible opportunities for emotional connection (30). The Belongingness and Love Needs Scale comprises 9 measurement items. Analysis using SPSS revealed a standardized reliability coefficient of 0.71 and a validity coefficient of 0.701, indicating satisfactory reliability and validity. Detailed data are presented in Table 7.

**Table 7:** Quantitative evaluation of belongingness and emotional support needs fulfillment.

No.	Question	Agreement	Mode	Mean score
1	Participating in group activities makes you feel happy.	73	4	3.97
2	The institution organizes various celebrations for different festivals to create a festive atmosphere.	67	5	4.03
3	Caregivers provide psychological support to help you maintain a positive mood.	34	3	2.87
4	The institution frequently organizes memory-sharing activities, allowing you to exchange life experiences with others.	80	5	4.1
5	Volunteers frequently visit and engage in activities with you.	24	3	2.9
6	You enjoy interacting with other residents, which makes you feel warmth and companionship.	24	3	2.77
7	The institution arranges for you and your family to participate in activities together (e.g., holiday dinners).	70	5	4
8	The institution offers various customized activities that match your interests (e.g., classroom learning).	10	1	2
9	Family members frequently call or visit to express their care.	24	3	2.9

‘Agreement’ refers to the percentage of respondents who rated an item with a score higher than 3 (i.e., a score of 4 or 5) on the 5-point Likert scale.

Statistical results demonstrate that the needs for belonging and love among the majority of older adults with dementia have not been adequately fulfilled. Combining interview data and quantitative analysis, the primary reasons are identified as follows:

1. The quantity of caregivers is insufficient. The daytime staff-to-resident ratio (1:4) is lower than the design standard (1:3) required by the “Zhejiang Provincial Implementation Plan for the Transformation of Dementia Care Zones 2023” leading to inadequate individual companionship.
2. Observations and interviews reveal that most older adults residents are not inclined to proactively communicate with their peers within the institution, as indicated by a peer interaction avoidance rate of 73%. Instead, they prefer to interact with visiting family members or volunteers. Nevertheless, the average frequency of family visits (2.4 times per month) is lower than the recommendation by the WHO (≥ 4 times per month), and the frequency of volunteer visits is unstable.
3. The institution lacks sufficient elements that reflect the residents’ previous professional and personal backgrounds (only 32% of rooms containing related items), thereby undermining their sense of belonging.

#### 3.1.4 Respect needs

Even with professional caregiving, cognitive decline may cause the older adults to feel neglected or devalued, which can erode their sense of self-identity and dignity (31). Considering their past roles as significant contributors within their families and society, as well as their extensive life experiences, they have a strong desire to be recognized as valuable and deserving of respect (32). Delivering personalized care, attentively listening to their concerns, and honoring their life habits and histories not only contribute to preserving their self-esteem but also strengthen their sense of belonging and security. This, in turn, fosters a more positive approach to life and enhances their overall quality of life (33). The Respect Needs Scale comprises 8 measurement items. SPSS analysis revealed a standardized reliability coefficient of 0.718 and a validity coefficient of 0.747, demonstrating robust reliability and validity. Detailed results are provided in Table 8.

**Table 8:** Quantitative evaluation of dignity and respect needs fulfillment.

No.	Question	Agreement	Mode	Mean score
1	Caregivers address you with your professional title (e.g., Teacher XX).	63	5	3.83
2	Caregivers provide you with private space when appropriate and do not disturb you.	44	2	2.6
3	Caregivers frequently encourage you and affirm your achievements.	67	4	3.87
4	You can choose what to wear each day.	67	4	3.87
5	The institution maintains your daily habits (e.g., post-meal walks, reading newspapers).	70	5	3.9
6	Caregivers ask for your consent before assisting you.	57	3	3.7
7	Caregivers remember your name and preferences.	63	4	3.73
8	Caregivers knock before entering your room.	24	3	2.67

‘Agreement’ refers to the percentage of respondents who rated an item with a score higher than 3 (i.e., a score of 4 or 5) on the 5-point Likert scale.

The data indicates that while the majority of older adults with dementia perceive that they are treated with adequate respect in their daily lives, some have expressed concerns about insufficient protection of their personal privacy. Furthermore, to facilitate management and ensure safety, caregivers often congregate the older adults in public areas and encourage their participation in social activities, thereby reducing their solitary time. As a result, some residents feel that their need for solitude has not been fully satisfied. Notably, in Question 2, the relatively high degree of agreement and the relatively low average score may reflect the disparities in the care management approaches and the quality of personnel across different floors.

#### 3.1.5 Self-actualization needs

The older adults with dementia have previously held certain roles in society, and as a result, they desire to express their self-worth through creative activities such as painting, music, and vocational reenactments (34). These activities serve to awaken long-term memories, while also affirming their capabilities and wisdom. Personalized activities and motivation tailored to their interests can help them regain a sense of purpose and accomplishment in life (35). Therefore, in caregiving practices, fostering self-actualization not only enhances their overall quality of life but also supports their psychological well-being. The Self-Actualization Needs Scale consists of 9 measurement items. SPSS analysis revealed a standardized reliability coefficient of 0.806 and a validity coefficient of 0.746, demonstrating high reliability and validity. Detailed data are provided in Table 9.

**Table 9:** Quantitative evaluation of self-actualization and personal growth needs fulfillment.

No.	Question	Agreement	Mode	Mean score
1	You have many opportunities to showcase your talents and creativity.	77	4	4.03
2	You actively respond to others’ care and attention.	70	5	3.93
3	Various social activities enhance your confidence in interacting with others.	73	5	4.13
4	Outdoor activities such as walking, Tai Chi, and gardening bring you inner fulfillment.	80	5	4.13
5	Activities such as painting, writing, and music help you express inner emotions.	67	5	3.93
6	The institution encourages you to develop personal hobbies, making your life more fulfilling.	76	4	4.07
7	You are willing to help other older adults residents with small tasks (e.g., passing objects).	67	4	3.67
8	You are willing to take part in planning daily activities (e.g., scheduling walking time).	27	2	2.5
9	The institution’s social activities provide you with more opportunities to connect with society.	54	3	3.67

‘Agreement’ refers to the percentage of respondents who rated an item with a score higher than 3 (i.e., a score of 4 or 5) on the 5-point Likert scale.

Statistical analyses reveal that the majority of older adults with dementia can fulfill their self-actualization needs relatively well. However, observational and interview data suggest that most of these individuals depend on caregivers for activity planning, exhibiting no explicit preferences, as reflected in the low agreement rate for participating in planning daily activities (Question 8: Agreement: 27%).

Despite this, they often engage actively in activities arranged by caregivers and derive both enjoyment and a sense of accomplishment, evidenced by high agreement on items like showcasing creativity (Question 1: Agreement: 77%) and finding fulfillment in outdoor activities (Question 4: Agreement: 80%). These findings imply that in the context of a decline in cognitive and self-decision-making capabilities, individuals are more likely to adopt a passive pattern of acceptance (40). For instance, during semi-structured interviews, when asked, “What kind of activities would you like to participate in?” even when researchers provided several options (such as dancing, walking, or puzzle games)—most respondents answered, “Anything is fine. I’ll do whatever is arranged.”

#### 3.1.6 Additional needs

Furthermore, the older adults with dementia articulated additional expectations regarding needs that were not encompassed by the questionnaire. These needs have been systematically categorized and are detailed in Table 10.

**Table 10:** Additional identified needs.

No.	Needs	Frequency
1	The desire for more young volunteers to provide companionship.	15
2	The wish for more frequent visits from family members.	11
3	A larger activity space.	2
4	The desire to find a companion.	3
5	A greater variety of activities.	2

Based on the other needs added by cognitive seniors, there is a large gap in the need to go for companionship for cognitive seniors.

### 3.2 Qualitative analysis

The data collected through field observations, questionnaires, and interviews were systematically integrated, with key terms, passages, and statements representing the needs of individuals with dementia being extracted and coded as variable factors. Subsequently, the encoded data were clustered and summarized to form categories. In conjunction with Maslow’s hierarchy of needs analysis method, the need themes were refined. The analysis was conducted using SPSS and NVivo software, with detailed results presented in Table 11.

**Table 11:** Categorization of needs among individuals with dementia.

No.	Key Statements	Categorization	Maslow’s hierarchy of needs	Theoretical association category
1	Flexible schedule	Schedule management	Physiological Needs	NDB model (Proximal environmental stimuli)
2	Quiet sleep/noise control	Environmental adaptation		
3	Mobility difficulties	Mobility support		NDB model (Background factors)
4	Inability to manage personal hygiene independently	Self-care support		
5	Poor vision and hearing	Sensory support		
6	Dietary preferences	Dietary adaptation		
7	Assisted eating	Tool adaptation		Kitwood theory (Individual preferences)
8	Health management	Health monitoring	Safety needs	NDB model (Background factors)
9	Cognitive training	Cognitive maintenance		
10	Imited movement	Autonomous activities		
11	Stable environmental space	Safety assurance		
12	Psychological comfort and support	Emotional support		Kitwood theory (Social psychology)
13	Personal solitude			
14	More diverse social activities	Social interaction	Belongingness and love needs	Kitwood theory (Social connection)
15	Unique holiday celebrations	Cultural interaction		
16	Additional outdoor activities	Environmental experience		NDB model (Proximal environmental enrichment)
17	Continuation of previous lifestyle habits	Historical continuity		Kitwood theory (Identity continuity)
18	Emotional care from caregivers	Emotional interaction		Kitwood theory (Person-centered care)
19	Visits and companionship from family	Family bonds		NDB model (Social interaction)
20	Emotional relationship needs (companionship)	Intimate relationships		Kitwood theory (Proximal needs compensation)
21	Involvement of younger generations	Intergenerational interaction		
22	Sharing life experiences	Historical expression		Kitwood theory (Role awakening)
23	Respect for privacy	Privacy management	Esteem Needs	Kitwood theory (Subjectivity)
24	Sufficient affirmation and support	Personality support		
25	Autonomy in daily life choices	Autonomous decision-making		Kitwood theory (Personalized care)
26	Assistance in life planning	Daily Living Support	Self-actualization needs	Kitwood theory (Creative activity intervention)
27	Relaxation through music and arts	Emotional expression		
28	Painting and writing			
28	Actively responding to external stimuli	Social interaction		NDB model (Proximal sense of meaning stimuli)
29	Expression of personal creativity	Talent demonstration		
31	Personal hobbies	Daily living support		Kitwood theory (Personhood continuation)
32	Individual expression	Individual uniqueness		

The comprehensive quantitative analysis results can lead to the conclusion that the older adults with dementia have needs in various physical and psychological aspects. While their physical needs are generally satisfied, certain indicators still remain insufficient. Comparatively, psychological needs attract more attention, and the data analysis indicates that their manifestations present certain differences from Maslow’s hierarchy of needs. Specifically, among the four types of psychological needs after physiological needs, the need of belonging and love is the most deficient, while the need of -actualization is relatively adequate.

## 4 Discussion

The special behaviors in the observation process of the older adults with dementia have been recorded and shown in the following Table 12.

**Table 12:** Observation record of special behaviors.

No.	Special Behavior	Frequency	Case	Theoretical association category	Theme
1	Wandering during nap time	4	3F1	NDB Model (Proximal environmental stimuli)	Behavioral compensation
2	Wandering with a water cup	2	3F2		
3	Hitting a caregiver	1	6F5		
4	Leaving in the middle of an activity	7	41F		
5	Dozing off during an activity	6	6F4		
6	Standing by the elevator door	3	6F1	Kitwood theory (Proximal need compensation)	Emotional compensation
7	Repeatedly inviting researchers for meals	12	8F1		
8	Bringing clothes to researchers	5	6F4		
9	Carrying luggage around	1	6F1		
10	Repeatedly talking about children	6	6F3		
11	Looking for a partner	3	5F1		
12	Guarding the room against researchers	3	7F1	Kitwood theory (Role awakening)	Privacy respect
13	Disregarding caregivers	3	5F3		
14	Being invited to teach dance	4	5F2	Kitwood theory (Role awakening)	Self-identity
15	Standing up when hearing military songs	2	5F1		
16	Refusing caregiver assistance	5	5F1	Kitwood theory (Social psychology)	
17	Apologizing repeatedly to others	1	2F1		
18	Refusing contact with others	2	6F2		

By synthesizing the previously identified need characteristics of individuals with dementia with their distinctive behavioral manifestations, and in light of the characteristics of the quantitative data, the Need-Driven Dementia-Compromised Behavior (NDB) model and Kitwood theory are employed for a more profound discussion, to explore the underlying causes of the need characteristics and offer targeted improvement strategies.

### 4.1 Interpretation of the need–behavior relationship through the NDB model

#### 4.1.1 Behavioral compensation for physiological needs deficiencies

The analytical framework of this study is grounded in Maslow’s hierarchy of needs; wherein physiological needs represent the most fundamental and persistent requirements for older adults with dementia. Quantitative data reveals that satisfaction levels for the midday rest environment and assistive walking tools are merely 15 and 23%, respectively, (Table 5), indicating that these unmet physiological needs constitute negative “background factors” that may directly trigger behavioral abnormalities in the “proximal environment.” Specifically, noise disturbances during midday rest could lead some the older adults to leave activities halfway (e.g., 4F1 returning to their room to sleep during an activity) or fall asleep during group events (e.g., 6F4 and 5F3 dozing off while singing), which might stem from sensory overload resulting in an imbalance of environmental stimuli (36). Likewise, the insufficiency of assistive walking devices constrains patients’ autonomous activities, potentially inducing frustration or resistance to caregiving interventions (e.g., 5F1, who is unable to stand up independently, resisting the assistance of caregivers during lunch). From the perspective of Kitwood’s “Person-Centered Care” theory, this resistance can be interpreted as an attempt to maintain personal control after their autonomy has been environmentally deprived.

Such behaviors validate the “need suppression → behavioral compensation” mechanism, through which individuals with dementia convey their needs via abnormal actions when their physiological needs (background factors) are unmet. This reveals the chained inhibitory effect of basic need gaps on higher-level psychological needs; that is, when foundational physiological needs are not satisfied, older adults with dementia are unable to pursue higher levels of safety, belonging, and esteem in a typical manner.

#### 4.1.2 Care resource deficiencies and delayed need response

The research reveals that a structural imbalance in care resources—specifically, the caregiver-to-resident ratio of approximately 1:4—is a core factor that constrains the efficacy of need response, which in turn can induce behavioral problems. The average daily working duration of up to 9 h means caregivers’ energy is primarily consumed by meeting basic physiological needs, leading to insufficient personalized interaction time (less than 2 h per person per day). This is reflected in the low satisfaction rate for emotional care provided by caregivers, which was merely 34% (Question 3 in Table 7). This imbalance between the supply and demand of care resources has given rise to anxiety and compensatory behaviors. For instance, 3F2 repeatedly wandered in the corridor due to an excessive delay in drinking water provision, and 5F1 refused care intervention because timely assistance for eating was not provided. The NDB model points out that “delayed proximal need response” will directly induces compensatory behaviors, and the research findings further corroborate this mechanism.

Although Kitwood’s theory advocates “personalized communication based on individual’s life history,” the high-intensity workload forces caregivers to adopt standardized processes (such as centralized medication administration and batch clothing changes), making it difficult to implement meticulous care (such as interaction designs targeted at the background of a former teacher like 5F1). This “de-personalized” process, driven by insufficient resources, effectively creates what Kitwood described as a “malignant social psychology” environment. Under these conditions, even if the institution intends to provide high-quality service, the anxiety and compensatory behaviors of the older adults with dementia can hardly be eliminated at their root as long as the fundamental issue of human resources remains unresolved.

#### 4.1.3 Privacy perceptions and the preservation of personhood

Quantitative data reveals that privacy needs (at the level of esteem needs) are systematically undervalued in institutional care: only 44% of residents consider their privacy to be fully respected (Table 8), and significant generational differences were manifested in interviews–younger caregivers (aged 26–30) are more inclined to protect privacy, while older caregivers (aged 41–60) insist that “safety takes precedence over privacy” (see question 8 in Table 8). In traditional culture, eldercare paradigm emphasizes “care without privacy”, placing collective management above individual rights. For instance, although keeping room doors open throughout the day and centralized activity monitoring cloud ensure the older adults’ safety needs (background factors of NDB), they deprive their personal space and autonomy (proximal needs). Kitwood’s theory points out that disregarding privacy essentially erodes “personhood,” and the older adults maintain their subjectivity through resistant behaviors (such as 7F1 cautioning others against approaching the room) or self-seclusion (such as 6F2 refusing to interact with others). The NDB model further discloses that when safety (background needs) and privacy (proximal needs) are imbalanced, older adults with dementia may fall into an “anxiety-withdrawal” cycle (37). Admittedly, when providing essential assistance such as personal hygiene to the older adults who are unable to manage these tasks independently, the boundaries of privacy can become blurred and difficult to navigate. However, this challenge does not justify the systemic disregard for privacy observed in institutional care.

#### 4.1.4 Creative activities and identity awakening

A seemingly contradictory yet highly insightful finding of this study is that despite a general deficiency in the mid-level need for “belongingness and love,” the older adults with dementia exhibit a relatively high degree of satisfaction with their self-actualization needs. Quantitative data reveal that creative activities, including painting and music, achieve a satisfaction rate of 70% (Table 9), with a significant proportion in the categories of “creative expression” and “personal hobbies” (Table 11). This phenomenon challenges the linear, hierarchical understanding of Maslow’s Hierarchy of Needs and reveals the unique nature of creative activities in dementia care.

This finding suggests that even in the context of cognitive decline, the older adults with mild to moderate dementia are capable of reinforcing their self-identity and sense of value through artistic endeavors. According to Kitwood’s theory, creative activities can evoke long-term memories via non-verbal channels (e.g., colors, melodies), thereby strengthening identity recognition and “personality persistence.” For example, veteran 5F1 demonstrated evident pride when recounting military experiences and instinctively stood at attention upon hearing a patriotic song, reflecting the supportive role of historical identity in maintaining self-worth.

The NDB model suggests that creative activities mitigate the sense of helplessness resulting from cognitive decline by fulfilling the “stimulus of meaning” within proximal needs. For instance, 5F2 (a former dance teacher) continuously laughed while instructing the researchers in dancing, revealing the positive modulation of artistic expression on the immediate emotional state. Therefore, for older adults with dementia, “self-actualization” is perhaps not an ultimate goal to be achieved only after satisfying all lower-level needs. On the contrary, creative activities centered on awakening identity function as a powerful therapeutic tool in their own right.

#### 4.1.5 Family bonds and emotional compensation

A core finding of this study is that the need for “belongingness and love” is the most severe and prevalent unmet need for older adults with dementia in institutional care. Evidence from semi-structured interviews and quantitative data reveals that satisfaction with care from family members was merely 24% (Question 9 in Table 7), and the frequency of unmet needs for family visits and companionship was 11 instances (Table 10), constituting a major point of conflict within the love and belonging dimension. The prolonged absence of family interaction causes patients to fall into a detrimental cycle of “feeling abandoned—self-doubt (38),” which directly gives rise to a series of compensatory behaviors aimed at combating separation anxiety and filling the emotional vacuum. For instance, resident 2F1 repeatedly apologized to everyone—including nursing staff, volunteers, and the families of other residents—because their daughter had not visited for five months; 6F1 consistently dresses neatly and waits at the elevator entrance daily for her children; and 6F3 meticulously prepares their appearance before visits, persistently inquires about their children’s whereabouts, and remains in a low mood afterward. All of these are ritualized actions to counter separation anxiety.

According to the NDB model, the insufficient frequency of family visits constitutes a proximal stimulus of “social interaction deprivation,” directly provoking agitated behaviors (e.g., repetitive inquiries about the whereabouts of children) and diminishing self-worth. Kitwood’s theory further elucidates that the disruption of family bonds accelerates the process of “social death,” compelling patients to reestablish social connections through attachment to objects (e.g., 6F1 carrying luggage) or emotional transference (e.g., 8F1 and 5F2 frequently inviting researchers to meals, 6F4 offering clothing as a friendly gesture), to forestall the disintegration of self-identity.

This “withdrawal–agitation” bipolar behavior (e.g., the coexistence of the withdrawn apology of 2F1 and the agitated inquiry of 6F3) markedly increases caregiving challenges, highlighting the exigency of social and emotional support. While the involvement of young volunteers temporarily bridges the companionship gap (Table 10), the absence of an institutionalized family interaction mechanism leaves emotional compensation fragmented and unable to fundamentally restore the hierarchy of belonging needs. Consequently, despite the fact that the higher-level need for self-actualization is largely satisfied through institutional activities (Table 9), the deficiency of fundamental family and social support still makes the need for belonging the key point of intervention.

#### 4.1.6 Cultural reshaping of need priorities

Quantitative analysis indicates that the self-actualization need presents a cultural paradox within this study: despite the high recognition rate for creative activities (80%, Table 9) and a considerable proportion of “creativity manifestation” (Table 11), qualitative interviews reveal that approximately 70% of respondents exhibit a passive acceptance attitude toward activity participation. For instance, Case 5F2 (a former dance teacher) is willing to showcase her talent only when invited, indicating that the expression of self-actualization is contingent upon external environment. Drawing on the NDB model, group belonging partially fulfills the need for meaning; however, the standardized nature of institutional activity arrangements constrains individual autonomy in self-expression.

This inconsistency becomes particularly evident in collective activities: while 73% of the older adults residents participate in institutional events (Question 1 in Table 6), a mere 27% actively express their personal preferences, reflecting the emphasis on group harmony in traditional Chinese culture (e.g., Case 6F4 participating in a dance that she was not interested in after being persuaded by the caregiver). This phenomenon aligns with social learning theory (39), suggesting that individuals who have long been influenced by collective culture have their behavioral choices shaped more by group norms rather than driven by personal will.

The core of these issues lies in the fact that Eastern culture prioritizes familial and collective identity, while the West attaches greater significance to individual autonomy. This cultural characteristic necessitates a re-examination of the study’s theoretical frameworks:

Reconstruction of Maslow’s Hierarchy: This reveals the cultural plasticity of the hierarchy. In an Eastern cultural context, the needs for “self-actualization” and “belongingness and love” are not distinct or mutually exclusive. Instead, “self-actualization” is often achieved by successfully fulfilling one’s role within the collective, thereby reinforcing the “sense of belonging.” Becoming a cherished and contributing member of the family and group is the highest form of value realization. Therefore, Case 6F3’s constant anticipation and ritualized preparations for her children’s visits are not merely a longing for affection but are more profoundly a confirmation of her core identity as a “mother “the very foundation of her self-worth.

Cultural Definition of the NDB Model’s “Stimulus of Meaning”: For this population, a “meaningful stimulus” is more likely to be something that involves “contributing to the collective” or “affirming important social relationships” rather than “gaining an opportunity for self-expression.” This also explains why the rupture of family bonds (termed “social interaction deprivation” in the NDB model) has such a significant negative impact —it directly threatens the primary source of the elders’ identity and life’s meaning.

Relational Interpretation of Kitwood’s “Personhood” Theory: In a Western context, Kitwood’s “personhood” is often closely linked to individual choice and uniqueness. However, in this study’s cultural context, “personhood” is more of a relational construct. An individual’s identity and value are largely defined by their position and relationships within family and social networks. Therefore, what best awakens and sustains their “personhood” is not encouragement to “go do what you want to do,” but rather activities that reinforce their key social roles (such as having a former teacher give a lesson or a veteran sing military songs).

In summary, the residents’ passive participation in activities and their intense yearning for family connection are two sides of the same cultural coin. In light of the findings on “Family bonds and emotional compensation,” it becomes clear that in a collectivist cultural context, the “sense of belonging” transcends other psychological needs to become the absolute core of maintaining self-identity. This finding has transformative implications for care practices: the focus of intervention should perhaps not be on providing more “choices,” but rather on creating more opportunities for the older adults to assume meaningful social roles, contribute to the collective, and strengthen their family connections.

### 4.2 Practical implications and strategies

Drawing on the “Hierarchical -Dynamic Need Response Framework,” this study proposes a series of systematic intervention strategies, with the aim of balancing the safety management and individualized care needs for older adults with dementia, filling the gap in the need for belonging and love, and responding to the influence of cultural differences on the priority of needs. The strategic framework is structured according to a progressive trajectory of “short-term optimization → medium-term integration → long-term reform,” integrating the NDB model’s behavioral compensation mechanism with Kitwood’s humanized care paradigm to achieve the transformation from theory to practice.

#### 4.2.1 Short-term: environmental and staffing adaptation

Among all intervention strategies, the highest priority should be given to adapting the physical environment and human resources, as satisfying physiological needs is the cornerstone for maintaining the emotional and behavioral stability of older adults with dementia. When basic needs such as physiological comfort are not met, older adults with dementia often express their distress through behaviors such as agitation, wandering, or resistance. Therefore, starting with optimizing the environment and supplementing resources can reduce the triggers for negative behaviors at their source, creating a stable foundation for subsequent higher-level interventions. The specific measures are as follows:

1. Environmental Transformation: Strengthen the sound insulation between various functional zones (e.g., improving door soundproofing) and standardize the auxiliary equipment repository (e.g., customized mobility aids and non-slip tableware) to effectively address the gaps in physiological needs.
2. Flexible Staff Scheduling: Enhance the deployment of caregiving manpower by dynamically reallocating staff during peak demand periods (e.g., activity periods after the midday rest). Under the condition of cost allowance, increase the caregiver-to-patient ratio from 1:4 to 1:3 to mitigate the chain behaviors triggered by untimely care.

#### 4.2.2 Mid-term: tripartite family-institution-society collaboration

Institutional activities can, to some extent, alleviate the sense of loneliness resulting from the absence of family. By participating in these activities, older adults with dementia can establish new emotional connections with caregivers and other residents or evoke fond memories of their families through specific themes, thereby achieving a form of psychological compensation. For example, one resident found emotional solace by making flowers for her daughter during a craft activity. However, the effectiveness of this substitute compensation is limited. Especially for residents with strong family attachments, such activities cannot truly replace the sense of belonging and identity derived from familial bonds. Therefore, the key to mid-term intervention design is to more closely integrate institutional activities with family participation.

1. Family Interaction Platform: Develop a family engagement calendar (e.g., countdown prompts for family visits) and a shared memory album (featuring historical family photographs) to mitigate separation-induced anxiety.
2. Develop a “Family-Institution” collaboration framework by organizing monthly “Family Day” events to encourage family participation in institutional activities.
3. Introduce a family point-based reward system to offer institutional service benefits (such as free health consultations) to families with frequent visits.
4. Intergenerational Volunteer Involvement: Partner with societal organizations such as higher education institutions and community groups to institutionalize youth volunteer programs, featuring intergenerational interaction activities (e.g., “Life Story Workshops”) to address emotional support deficiencies of caregiving practices.
5. Unite families and community, encourage diversified forms of care (such as shooting short videos or audio messages).

#### 4.2.3 Long-term: vocational reenactment & personalized profiling

After satisfying the basic physiological and emotional needs of the older adults with dementia, the focus of long-term intervention shifts to rebuilding their personal dignity and transforming the institution from a mere ‘place of residence’ into a genuine ‘home.’ When the institutional environment lacks sufficient elements that reflect the residents’ previous professional and personal backgrounds, their sense of belonging is directly undermined. Personalized interventions based on “life stories,” which awaken long-term memories related to personal history, can effectively strengthen their “personhood” and combat the dissolution of self-identity. Therefore, more in-depth, personalized strategies must be implemented to help older adults with dementia make the leap from merely “surviving” to truly “living.”

1. Occupational Reinstatement: Customize intervention plans based on the historical backgrounds of individuals, such as curating a “Red Song Choir” for veterans or hosting a “Story Salon” for retired educators, thereby fostering self-actualization through identity awakening.
2. Personalized Interest Archive: Establish individualized interest inventories (such as gardening and calligraphy), match activity resources and update them dynamically to ensure that more than 70% of the older adults participate in interest-related activities.
3. Cultural Adaptation: In light of the collective cultural background, design the “Compatible Creativity Stimulation” projects. For instance, embed individual theme creation (e.g., 5F2 dance choreography) within group collaboration (e.g., group singing relay) to balance group harmony and individual expression.
4. Implement a “Need-Behavior” correlation framework to train caregivers to identify unmet needs via behavioral analysis. Train caregivers with techniques for delivering “acknowledgment responses” (e.g., “Your daughter is coming tomorrow. She asked me to accompany you first”).

## 5 Conclusion

This study synthesizes Maslow’s Hierarchy of Needs Theory, the NDB model, and Kitwood’s Person-centered Care Theory to construct a “Stratified - Dynamic Demand Response Framework,” and puts forward strategies such as short-term environmental optimization, mid-term social support linkage, and long-term personalized care, in order to enhance the sense of social connection and psychological well-being of older adults with dementia. Specifically, the study arrives at the following main conclusions:

1. The Need Gap for Belonging and Love: Although institutions are capable of fulfilling the majority of physiological and safety needs of older adults people with dementia, their family and social emotional needs are frequently disregarded, resulting in an escalation of loneliness and exerting adverse effects on mental health.
2. Cultural Influences on the Prioritization of Needs: Eastern collectivist cultural orientation fortifies the sense of group belonging while attenuating individual autonomy, rendering the older adults more reliant on institutional arrangements rather than proactively expressing their individualized needs. This characteristic contrasts with the Western model that places emphasis on individual autonomy.
3. The Dynamic Correlation Mechanism between Needs and Behaviors: In accordance with the NDB model, when needs remain unfulfilled, the older adults with dementia typically manifest as behavioral compensations. For instance, overly prolonged waiting for care may give rise to anxious wandering, and insufficient family visits may trigger emotional compensatory behaviors, signifying that the degree of demand satisfaction directly influences the behavioral patterns of the older adults

The Significance of Personalized Care: Personalized interventions such as occupational reenactment and interest matching can effectively enhance the self-identity and psychological well-being of the older adults with dementia. Nevertheless, the standardized care model employed by institutions often overlooks individual differences, leading to a situation where some the older adults participate in activities but lack genuine interest-driven impetus.

The Proposal of Stratified Intervention Strategies: This study has constructed a “Stratified - Dynamic Need Response Framework” and put forward strategies such as short-term optimization (e.g., adjustment of care resources, upgrading of assistive devices), medium-term integration (e.g., incentives for family visits, volunteer companionship), and long-term intervention (e.g., occupational reenactment, interest profiles) in order to more accurately meet the needs of older adults with dementia.

Nevertheless, this study presents certain limitations. First, the research sample is confined to a specific care institution in Eastern China, which may entail regional cultural biases, thus being unable to comprehensively represent the need characteristics of older adults with dementia across diverse regions and institutional types. Second, the official data collection phase was concentrated within a 16-day period. Although a friendly rapport was established with participants through preliminary engagement, we acknowledge that this relatively short duration may potentially impact the depth of the qualitative data and the generalizability of the findings.

The research methods primarily involve interviews, observations, and questionnaire surveys. Although they can effectively reflect the current scenario, their cross-sectional nature limits the ability to track long-term trends in residents’ needs. Future research could employ longer, longitudinal observations to more comprehensively capture these dynamic changes. Finally, the work pressure and practical operational constraints of caregivers mean that many theoretically feasible intervention measures still face challenges during actual implementation. For example, how to strike a balance between privacy protection and personalized care while ensuring safety still requires further deliberation in future practice.

## Data Availability

The original contributions presented in the study are included in the article/supplementary material, further inquiries can be directed to the corresponding author/s.
